# Immune Pathogenesis of COVID-19 Intoxication: Storm or Silence?

**DOI:** 10.3390/ph13080166

**Published:** 2020-07-26

**Authors:** Mikhail Kiselevskiy, Irina Shubina, Irina Chikileva, Suria Sitdikova, Igor Samoylenko, Natalia Anisimova, Kirill Kirgizov, Amina Suleimanova, Tatyana Gorbunova, Svetlana Varfolomeeva

**Affiliations:** FSBI N.N. Blokhin National Medical Research Center of Oncology, Ministry of Health of Russia, 24 Kashirskoye sh., 115548 Moscow, Russia; irinatchikileva@mail.ru (I.C.); suriyasitdikova@yandex.ru (S.S.); i.samoylenko@ronc.ru (I.S.); n_anisimova@list.ru (N.A.); kirgiz-off@yandex.ru (K.K.); aminasuleymanova313@gmail.com (A.S.); wasicsol@mail.ru (T.G.); varfolomeeva-07@mail.ru (S.V.)

**Keywords:** COVID-19, “cytokine storm”, acute respiratory distress syndrome (ARDS), macrophage activation syndrome, lymphopenia, immuno-paralysis

## Abstract

Dysregulation of the immune system undoubtedly plays an important and, perhaps, determining role in the COVID-19 pathogenesis. While the main treatment of the COVID-19 intoxication is focused on neutralizing the excessive inflammatory response, it is worth considering an equally significant problem of the immunosuppressive conditions including immuno-paralysis, which lead to the secondary infection. Therefore, choosing a treatment strategy for the immune-mediated complications of coronavirus infection, one has to pass between Scylla and Charybdis, so that, in the fight against the “cytokine storm,” it is vital not to miss the point of the immune silence that turns into immuno-paralysis.

## 1. Introduction

**Figure 1:**
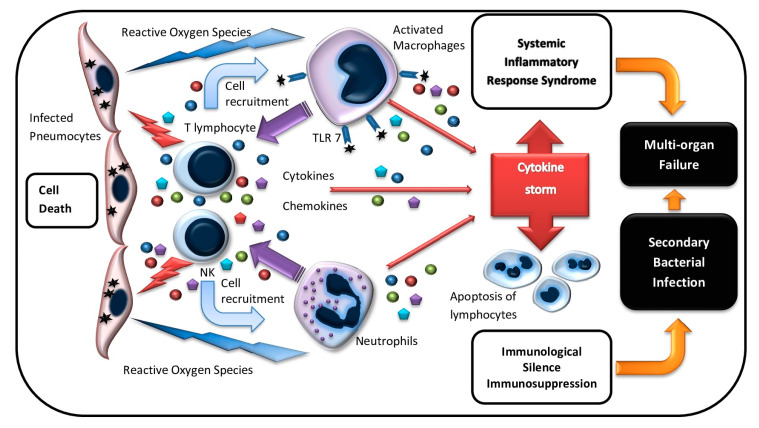
Immunologic damage with COVID-19 infection. Pneumocytes infected with SARS-CoV-2 are recognized and lysed by innate (NK cells) and adaptive (T cells) immune effector cells. Activated lymphocytes produce a wide range of cytokines and phagocyte recruiting chemokines, which attract macrophages and neutrophils to the infection site. Activated macrophages and neutrophils release reactive oxygen species (ROS) that damage lung tissue. Macrophages are also induced via TLR7 interaction with viral RNA. Macrophage stimulation triggers pro-inflammatory cytokine overproduction and the “cytokine storm,” which results in systemic inflammatory response syndrome and multiple organ failure. Increased level of inflammation mediators leads to the apoptosis of immune effector cells, which causes lymphopenia and, subsequently, immunosuppression. Secondary bacterial infections may develop at this stage.

COVID-19 (corona virus disease 2019) was first officially registered in China in December 2019 and it is one of the most widespread and aggressive infection along with SARS (severe acute respiratory syndrome) and MERS (Middle East respiratory syndrome) [1,2]. According to the COVID-19 open datasets (e.g., Kaggle COVID-19 Dataset) by July 2020, there were more than 14.5 million confirmed COVID-19 cases and a mortality rate of 608,000 worldwide. In 2002, approximately 8000 people were infected by SARS-CoV (severe acute respiratory syndrome-related coronavirus) in Guangdong province in China with a mortality rate of 10%. In 2012, the second infection outbreak was caused by MERS-CoV [3]. Those three coronaviruses SARS-CoV, MERS-CoV, and SARS-CoV-2 caused dry cough, distress, myalgia, and headache. Patients with severe disease developed marked severe acute respiratory syndrome and respiratory distress syndrome (ARDS) [4,5,6,7,8]. The immune system plays an important role in the fight against viral infection. However, an excessive immune response can lead to a systemic inflammatory reaction, organ disorders, and multiple organ failure (MOF) (Figure 1). An excessive or aberrant immune response can mediate the development of atypical pneumonia. This connection is proved by the fact that the grade of respiratory distress increases in spite of the decline of the viral load [9]. In addition, the progression of ARDS is associated with the increase of circulating inflammatory mediators that induce such dangerous COVID-19 complications as a systemic inflammatory response and sepsis-like conditions [10,11,12]. At the advanced stages of the disease, patients may develop pulmonary failure and MOF with acute liver and kidney dysfunction, hypoxic encephalopathy, disseminated intravascular coagulation/thromboembolic disease (DIC), and septic shock. The development of these complications is closely associated with fatal outcomes in COVID-19 patients. Therefore, this infection can cause both ARDS and systemic inflammatory reactions that lead to MOF. The “cytokine storm,” which can induce a systemic inflammatory response, virus-associated sepsis, inflammatory lung disorders such as pneumonitis or ARDS, shock, and organ or MOF plays a significant role in the COVID-19 pathogenesis, which is similar to that shown for SARS and MERS (Figure 1). Another symptom of a severe coronavirus infection is lymphopenia, which can become critical at the advanced stages of the disease [13,14].

So far, no effective specific antiviral treatment for COVID-19 has been presented. Therefore, supportive therapy that relieves the symptoms and maintains functions of many organs can be especially helpful. Antiviral agents such as Oseltamivir, Arbidol, Lopinavir/Ritonavir, and Interferon-α demonstrated a certain effect in mono- or combination therapy. However, glucocorticoid therapy aimed at curbing the “cytokine storm” did not show any registered clinical effect. Immunoglobulin therapy has also shown low effectiveness. Thus, identifying high-risk population and/or starting treatment as soon as possible are crucial for reducing the death rate.

According to the MEDLINE database (PubMed) in 2020 (as of July 2020), there were more than 150 clinical trials and reviews published that described the immune system disorders, which are the major part of the coronavirus pathogenesis, in COVID-19 patients.

However, the immune mechanisms that induce a systemic inflammatory response, organ failure, and multiple-organ failure in patients with COVID-19 are still unknown. Thus, a better understanding of the immune disease pathogenesis is of ultimate importance for providing the scientific basis to achieve the effective treatment of the infection. We thematically focus on the immunopathogenesis of COVID-19 and related CoVs, clinical studies, and prognostic immunological markers as well as current and prospective immunotherapeutic strategies.

## 2. COVID-19 in Cancer Patients

It is assumed that COVID-19 infection implies an increased risk for cancer patients, especially those who have recently received chemotherapy, radiotherapy, and immunotherapy [15]. Liang W. et al. [16] demonstrated that cancer patients had a higher risk of developing severe complications of coronavirus infection, which require invasive ventilation as well as a higher mortality rate, compared with non-cancer patients. In addition, patients who received chemotherapy or surgery within a month had a higher risk of developing a severe disease, compared to cancer patients who did not receive anti-tumor treatment. Similar data were received in a retrospective multicenter study that included 67 cancer patients with COVID-19 from hospitals in Wuhan, Hubei province, China. The study showed that a larger part of cancer patients with COVID-19, as compared to non-cancer patients, developed severe disease and had a poor prognosis. The tendency for poor prognosis was more clear in patients during the course of anti-tumor treatment [17]. Hospitalization and repeated hospital visits were also found to be a potential risk factor for developing COVID-19 [18]. On the whole, 48–54% of cancer patients were at a higher risk of severe events and from 5.6% to 29% of cases were at risk of death [19]. Yet, it is interesting to note that patients with lung cancer did not have a higher risk of severe complications when compared to other cancer types. Cancer patients receiving immunotherapy with checkpoint inhibitors may also have an increased risk of severe complications of coronavirus infection as a result of immune-mediated events caused by the “cytokine storm”. This observation demonstrates the similarity of the mechanisms of immune dysfunctions, which results from COVID-19 complications and anti-tumor therapy with immune checkpoint inhibitors (ICI) [20]. However, the susceptibility to bacterial or viral infections in cancer patients with ICI therapy has not been studied widely enough. On the one hand, unlike chemotherapy, ICI immunotherapy can reconstitute cell immunity competence [21]. However, on the other hand, there are concerns about ICI treatment of patients with COVID-19 since ICI-induced pneumonitis can exacerbate viral pneumonia. The effects of over-production of ICI-induced inflammatory mediators and viral infection are likely summed up, which can lead to fatal consequences [22]. It is also assumed that adoptive immunotherapy by genetically modified CAR-T cells (chimeric antigen receptor T cells) increases the risk of a poor outcome in cancer patients with COVID-19, while taking into account the fact that the “cytokine storm” is a frequent unfavorable effect of CAR-T cell therapy [23]. Immunodeficiency that occurs in children with cancer receiving antitumor treatment may present potential risks of severe COVID-19 complications. However, a few reported cases of COVID-19 in children receiving anti-tumor treatment were described as asymptomatic or displayed mild symptoms [24]. Moreover, some data showed that patients, which include both adults and children with suppressed immune systems as a result of developing malignancies, antitumor therapy, and transplantation, achieved a favorable effect in treating COVID-19 when compared to patients with other concomitant diseases. Such results can be explained by the lack of overexpression of the inflammatory reactions due to the suppressed immune response, which determines the weak manifestation of the disease caused by SARS-CoV-2 [25].

Despite the rare combination of cancer and COVID-19 in children, it is worth paying closer attention to two patient groups—children in the first year of life and children with acute lymphoblastic leukemia (ALL) who are receiving supportive therapy. Limited follow-up experience registered lethal outcomes in both groups. Probably, these poor results were determined by severe immune dysfunction, which was caused, on the one hand, by the age-related immaturity of the immune system and, on the other hand, by the failure of the lymphocytic arm of the immunity. Italy has its peculiar experience in the treatment of children with cancer and following hematopoietic stem cell transplantation with concomitant coronavirus infection. Thus, Balduzzi A. et al. described a relatively mild course of COVID-19 in children with acute lymphoblastic leukemia, osteosarcoma, and rhabdoid tumor [26]. The patients included in the study had a standard risk of the disease. Therefore, the authors made a conclusion of mild COVID-19 infection in this cohort [26]. The study of Liang W. et al. analyzed 18 patients with COVID-19 and a history of cancer [16]. The authors demonstrated a more severe course of COVID-19 in cancer patients and, as a result, a worse outcome of the disease. Different scientific articles describe general measures of prevention and treatment of COVID-19 in children with cancer, which correspond to those recommended for the anti-infectious treatment of general population [27]. However, the authors suggest continuing the therapy as complete as possible, but refraining from intensive chemotherapy and especially immunotherapy for children with cancer in remission.

## 3. Lymphopenia—As a Prognostic Marker for the Severity of COVID-19 and Immunosuppression

Lymphopenia indicates the grade of the disease progression. It was registered in 85% of patients with COVID-19 in the critical condition, which provides the most significant prognostic value regarding other laboratory test characteristics [28]. The predicting parameter may be considered either relative or an absolute number of lymphocytes in the peripheral blood. The relative lymphocyte number in patients with severe viral intoxication went down to 5% within two weeks after the onset of the disease and increased to about 10% while recovering. However, patients with moderate intoxication had only minor deviations of this parameter [29]. The declining relative number of lymphocytes to levels lower than 20% patients with COVID-19 within the first 10–12 days of the disease is considered an unfavorable prognostic factor, and a decrease in lymphocytes below 5% is associated with a high risk of death and these patients need intensive therapy. Patients in the intensive care units had an average lymphocyte count of 800 cells/µL. In severe cases, the absolute lymphocyte number fell dramatically to 100 cells/µL [30,31]. As a rule, persistent and severe lymphopenia was observed in deceased patients in contrast to those who recovered. These data suggest that intoxication with COVID-19 leads to disorders of the immune status of patients, which causes immunosuppressive conditions and can result in immune paralysis. Though the majority of patients with coronavirus had lymphopenia, they had no significant changes in the neutrophil count in the blood. The enhanced ratio of neutrophils to lymphocytes (N/L) was observed in patients with severe grade of the disease and lymphopenia, which is considered a clear marker of systemic inflammation. Thus, in patients with sepsis, a high N/L ratio correlated with the severity of the disease by the APACHE II scale and the 28-day mortality rate [32]. These data suggest that the increased N/L ratio is associated with a poor outcome in patients with COVID-19.

Patients with COVID-19, especially with the severe events, developed lymphopenia mainly as a result of decreasing CD4^+^Th (T helper) cell number. However, CD8^+^T-cell and B-cell numbers showed no significant change. Marked lymphopenia was associated not only with the reduced CD3^+^ CD_4_^+^T-cell numbers, but also with the inhibition of their differentiation to effector memory cells from naive CD4^+^ T-cells, which play a particularly important role in the adaptive anti-infectious immunity. It is well known that the balance between naive CD4^+^ Th cells and memory T-cells is crucial for developing an effective immune response [33]. Patients with severe COVID-19 had a decreased T-regulatory (Treg) cell number, which play a key role in attenuating the excessive inflammatory response to viral infection [34,35,36]. The authors report that, in patients with COVID-19, they observed the enhanced expression of T-cell activation markers such as CD69 and CD38 that play an important role in clonal expansion of lymphocytes and production of various cytokines including pro-inflammatory ones [37,38,39]. In particular, the previous studies have found that activated T-lymphocytes have the increased production of IL-2, IFN-γ, and high levels of intracellular cytokines, such as IL-6 and GM-CSF (granulocyte-macrophage colony-stimulating factor), which play the major role in the induction of the “cytokine storm” in coronavirus infections [40,41,42,43]. On the other hand, some authors showed that Treg cells expressing activation markers CD38 and CD69 were characterized by increased suppressor activity and, therefore, enhanced IL-10 production as compared to naive Tregs [44]. Similar to the conditions in patients with sepsis, the decrease of HLA-DR expression on CD14^+^ monocytes, which downregulates their antigen-presenting function and prevents induction of the adaptive immune response, represents an indication of the severe immunosuppressive conditions that lead to immune paralysis in patients with COVID-19 [45].

These data demonstrate the dysregulation of the immune reactions in patients with COVID-19 at the critically severe stage that emerge as excessive production of both pro-inflammatory and suppressive bio-regulators, which mostly determine the immune pathogenesis of the infection (Figure 1).

## 4. Cytokine Storm

Cytokines and chemokines are especially important factors for down-regulation or up-regulation of the immunity and immunopathology in viral infections [46]. Although there is no direct evidence of pro-inflammatory cytokines and chemokines in lung pathology of patients with coronavirus infection, changes in laboratory test results, such as elevated serum levels of cytokines and chemokines in the infected patients, correlated with the disease severity grades and poor outcome, which reveals the role of excessive inflammatory reactions in the COVID-19 pathogenesis [47]. In particular, circulating IL-6 levels are closely associated with the severity of COVID-19. Elevated IL-6 concentrations were observed in patients with severe respiratory distress syndrome. Ulhaq ZS et al. [48] performed a meta-analysis of nine studies and showed that, despite the heterogeneity of the included cohorts, IL-6 levels were significantly higher in patients with severe events compared to patients with mild or moderate grades of the disease severity. Excessive IL-6 production, as shown before, leads to inhibition of the HLA-DR (human leukocyte antigen – DR isotype) surface expression on CD14^+^ monocytes [49]. Likely, the same processes occur in patients with COVID-19 and, thus, IL-6 overproduction mediates low HLA-DR expression on CD14^+^ monocytes.

The enhanced concentrations of the soluble receptors to IL-2, IL-8, IL-10, and TNF-α (tumor necrosis factor alpha) were registered in addition to the increased levels of IL-6 in serum [47], while IL-1β was not determined in most patients [12]. C. Huang et al. studied cytokines and chemokines in patients with COVID-19 and showed that higher concentrations of IL-2, IL-7, IL-10, G-CSF (granulocyte colony-stimulating factor), IP10 (INF-γ-inducible protein 10), MCP1 (monocyte chemo-attractant protein 1), MIP1A (macrophage inflammatory protein 1 alpha), and TNF- α were registered in the plasma of patients with severe grades of infection [50]. Zhou Y. et al. [41] analyzed the immune parameters of patients with COVID-19, especially those with severe infection and poor outcome, and showed that they also had elevated serum concentrations of inflammation-related cytokines including IL-2, IL-7, IL-10, G –CSF, IP10, MCP1, MIP1A, and TNF-α. Some authors mentioned about over-production of other mediators of inflammation such as IL-12, IL-13, IL-17, IFN-γ, MCP1, hepatocyte growth factor (HGF), TNF-α, and vascular endothelial growth factor (VEGF) [51].

An excessively activated immune response could be caused by pathogenic GM-CSF+Th1 cells and by the induced or inflammatory CD14^+^ CD16^+^ monocytes/macrophages, which play an important role in pulmonary immunopathology and systemic inflammatory reaction in patients with COVID-19. The enhanced levels of pro-inflammatory bio-regulators in the infected patients led to activating Th1 pathway of the cell immune response. However, COVID-19 infection also initiated increased secretion of Th2 cytokines such as IL-4 and IL-10 [52]. Thus, these results demonstrate that determining the rates of circulating cytokines, and, in particular, IL-6 and GM-CSF, may be of ultimate importance for prognosis of the disease progression in patients with COVID-19. In addition, inflammatory mediators are potential targets for the anti-infectious therapy of COVID-19.

## 5. Immunopathogenesis of Acute Respiratory Distress Syndrome and Systemic Inflammatory Response Including Macrophage Activation Syndrome

At the first stage of viral infection and in the course of the entire infectious process, the virus clearance involves innate immune effectors, NK (natural killer) cells, and γδT-cells that can recognize and lyse virus-infected cells skipping the stage of antigen presentation as well as recruiting neutrophils, monocytes, and macrophages to the infection-related site via cytokine and chemokine production (Figure 1). On the one hand, the attraction of various innate immune cells to the infection site provides virus elimination and triggers the adaptive immune reactions by antigen presentation. However, these immunocytes can have a secondary damaging effect, which releases a large number of active mediators and reactive oxygen species. The active mediators and reactive oxygen species destroy not only virus-infected cells but also the adjacent intact cells (Figure 1).

Excessive production of bioactive molecules can induce a systemic inflammatory response, organ failure, and MOF, and cause immunosuppression as a result of lymphocyte apoptosis. Along with that process, the developing lymphopenia, mainly as a result of Th cell activity, prevents the generation of an adaptive immune response while virus elimination by the innate immune effectors is ineffective because of their non-specific mechanism of action. The increased IL-6 level additionally impairs natural killer (NK) functions [53].

The symptoms of COVID-19 in the infected patients range from fever, headache, fatigue, etc. to DIC-syndrome, shock, and MOF. The development from mild to severe disease in patients with COVID-19 may be caused by a “cytokine storm”, which is mainly determined by macrophage activation. Macrophages are key producers of cytokines including IL-6 and other inflammatory mediators in response to viral infections that induce “cytokine storm” and systemic inflammatory reactions [54]. Macrophage activation syndrome (MAS) is described in detail for patients with autoimmune diseases such as juvenile idiopathic arthritis and systemic lupus erythematosus [55].

Patients who develop MAS have a rapid onset of fever, cytopenia, coagulopathy, increased transaminases, and MOF, i.e., the clinical picture is similar to the intoxication caused by COVID-19. One of the main MAS symptoms is hemo-phagocytosis, which is absorption of blood cells, including erythrocytes, leukocytes, or platelets in the bone marrow by liver and spleen CD163^+^ macrophages and/or histiocytes expressing the highly affine scavenger-receptor complex hemoglobin-haptoglobin [56]. Soluble alpha chain of IL-2 receptor (sCD25) and sCD163 in the serum are the symptoms of hemophagocytosis [57]. One of the macrophage activation syndrome manifestations is a high level of inflammatory mediators, such as IL-6, IL-1β, IL-18, and IFN-γ [58,59]. The examination of patients with MAS revealed inflammatory infiltrates consisting mainly of activated T-lymphocytes and macrophages or histiocytes that engulf normal hematopoietic cells [60]. Hemophagocytic lymphohistiocytosis or MAS in patients with autoimmune diseases is a severe complication and is characterized by a sepsis-like clinical picture [61]. Historically, the gold standard for MAS treatment has been glucocorticoids, immunoglobulins, and cyclosporin. However, despite the intensive therapy, the mortality rate of these patients reaches 20% [62]. MAS or hemophagocytic syndrome develops in cases of severe viral infections with fever. In particular, hemo-phagocytosis was observed in Dengue fever with MOF syndrome and Epstein-Barr virus induced infection. MAS manifestation in viral infections is detected as a systemic inflammatory reaction resulting from excessive activation of lymphocytes and macrophages. Uncontrolled immune over-activation is associated with a high temperature, cytopenia, coagulopathy, and increased sCD25 and sCD163 serum levels [63,64]. The symptoms of hemo-phagocytosis by giant multinuclear macrophages were registered in patients with weak immunity, which is susceptible to pneumonia caused by various viral infections [65]. Giant macrophages and hyperplastic pneumocytes were found in patients with SARS and diffuse alveolar lung impairment. Such giant cells were not found in the lung biopsy samples of patients at the terminal stage and were detected only when the disease was progressing for more than eight days [66]. The events of hemophagocytosis by macrophages in the lungs and the atrophy of the white pulp of the spleen were revealed in patients with SARS. The same morphological changes were also observed in patients with the H5N1 influenza with poor prognosis in 1997. The researchers considered that hemo-phagocytosis was caused by cytokine dysregulation in H5N1 influenza pneumonia [4,67,68]. Similar morphological changes in the lungs were found by Chinese researchers in biopsy samples of patients with COVID-19. The authors noted the infiltration of lung tissue by CD_68_^+^ macrophages [69]. Rossi F. et al. reported that all patients with COVID-19 and symptoms of severe respiratory failure (SRF) developed excessive inflammatory reactions with signs of immune dysregulation or MAS after M1 macrophage activation by pro-inflammatory mediators [70]. However, more information is required to clarify the role of M1 and M2 macrophage subpopulations.

Many doctors consider intoxication associated with severe COVID-19 as a sepsis-like condition, which is similar to the process associated with bacterial pneumonia with a fatal outcome [71]. However, some authors suggest that COVID-19 has a unique pattern of immune dysfunction, which manifests as lymphopenia, higher C-reactive protein levels, and increased D-dimers that represent the imbalance between pro-inflammatory and anti-inflammatory cytokines and, perhaps, this is what distinguishes this infection from classic sepsis [72]. However, immune dysregulation in classic sepsis in critically ill patients also manifests as MAS and a “cytokine storm” [73]. Additionally, sepsis-induced immuno-paralysis is characterized by lymphopenia, mainly through CD4^+^ T-lymphocytes [74,75], which makes it possible to consider severe COVID-19 intoxication as a sepsis-like syndrome or viral sepsis.

## 6. Perspective Approaches for Treating the “Cytokine Storm” and Immunosuppressive Conditions

Due to the large number of cytokines induced by SARS-CoV (severe acute respiratory syndrome coronavirus) and MERS-CoV (Middle East respiratory syndrome coronavirus) infections, corticosteroids have often been used to treat patients with severe diseases to diminish lung parenchyma impairment resulting from an excessive inflammatory reaction. However, the accumulated data suggest that, in patients with SARS and MERS, corticosteroids did not have any impact on the mortality rate, but rather slowed down the virus clearance [54]. COVID-19 patients with ARDS and MOF have elevated levels of IL-6 and C-reactive protein, which are characteristic indications of these severe conditions [53]. Novi G. et al. [76] described a case of COVID-19 in a patient with systemic scleroderma associated with B-cell depletion caused by Ocrelizumab therapy. The patient had no serious complications connected with the condition of the induced immunosuppression. The authors suggested that B-cell depletion could have played a beneficial role as a result of the decreased IL-6 production, generally released by peripheral B-cells.

The available data present the potential effect of IL-6 and IL-6-receptor blocking antibodies to combat the “cytokine storm” [77,78]. Thus, Tocilizumab (TCZ), which is a recombinant humanized IL-6 receptor monoclonal antibody, has been recommended for treating patients with COVID-19 at risk of a “cytokine storm.” TCZ has demonstrated efficacy in treating a steroid-resistant cytokine release syndrome in cancer patients treated with immune checkpoint inhibitors [79]. IL-6 is one of the most significant cytokines involved in the COVID-19-induced “cytokine storm.” For this reason, TCZ is recommended for severely ill patients infected with a new coronavirus infection with elevated IL-6 levels [80]. Clinical results of the studies in small cohorts showed that TCZ could be proposed as a promising therapeutic strategy for severely and critically ill patients with COVID-19. Nevertheless, more controlled studies are necessary to evaluate the reliable clinical efficacy of Tocilizumab in patients with COVID-19 infection. In addition, it should be taken into account that immune suppressive agents, such as corticosteroids or JAK inhibitors (Janus kinase inhibitors) used for suppression of the excessive inflammatory reaction, may increase the immunosuppressed patient’s condition and lead to inhibition of antiviral and antimicrobial immunity [81,82]. The in vitro studies showed that the immune suppressive JAK inhibitor Tofacitinib inhibited the IFN-α production [83]. Suppression of the interferon or other mediators (e.g., IL-6) may also mediate the development of the secondary bacterial infection along with the immune suppression [84]. Therefore, the use of immunosuppressive agents in critically ill patients with COVID-19 to decrease the inflammatory reactions should be carefully worked out considering possible inhibition of antimicrobial immunity.

Methods of extracorporeal blood purification therapy are effective for eliminating inflammation mediators in sepsis and a systemic inflammatory response syndrome [85]. The effectiveness of this approach was demonstrated in two patients with COVID-19 who underwent plasma exchange and CRRT (continuous renal replacement therapy) with an oXiris ^®^ hemofilter and a modified AN69 surface-treated membrane with adsorption capacity. The authors noted the decrease of IL-6 and the C-reactive protein after the procedure and improvement of the patients’ general health status [86]. Another study evaluated CRRT effect performed in five patients before they were registered as COVID-19 positive. The authors suggested that CRRT played an essential part in treating COVID-19 with chronic renal disease. If the symptoms of acute kidney injury appear, potential interventions, including CRRT, should be used to protect renal function as early as possible [87]. According to the researchers, CRRT can have a therapeutic effect on COVID-19 intoxication by removing potentially harmful toxins and stabilizing the patient’s metabolic and hemodynamic status [88]. Extracorporeal blood purification can eliminate inflammatory mediators and control the “cytokine storm” at an early stage of intoxication. However, randomized studies are required to prove the effectiveness of extracorporeal blood purification.

Mesenchymal stem cells (MSCs) can alter the cytokine secretion profile of macrophages, dendritic cells, naive and effector T-cells (Th type 1 and 2), and natural killer cells (NKs), which induces an anti-inflammatory or tolerant phenotype. In particular, MSCs inhibited production by mature dendritic cells (DCs) of pro-inflammatory cytokines such as TNF-α and IFN-γ and boosted the secretion of anti-inflammatory cytokines such as IL-10 and IL-4. MSCs also had an impact on the enhancement of the suppressor subpopulation of Tregs in the peripheral blood [89,90]. MSCs demonstrated promising effects in experimental ARDS model, which neutralizes the alveolar collapse, collagen accumulation, and cell apoptosis in the lung tissue. Several randomized phase 1 clinical trials confirmed the safety of MSCs obtained from various sources at a single dose of 1 million cells/kg to 10 million cells/kg in patients with ARDS of non-viral origin [91,92,93]. MSCs are considered as a potential therapeutic strategy for treating steroid-resistant MAS arthritis patients [94].

Clinical efficacy of peripheral blood MSCs was shown in patients with influenza A (H7N9) with ARDS. In particular, it was shown that the mortality rate was 17.6% in the group of patients who received MSCs versus 54.5% in the control group. The authors believe that these data demonstrated the potential of MSC-based therapy of patients with COVID-19 since the complications caused by these viruses have common mechanisms [95].

Other authors have recently published the results of a pilot study of seven patients with COVID-19-related pneumonia where they showed that clinical symptoms of all the patients improved significantly two days after MSCs transplantation. The study found the increased lymphocyte number in peripheral blood and the disappearance of activated subpopulations of CXCR3^+^ CD4^+^ T-cells, CXCR3^+^ CD8^+^ T-cells, and CXCR3^+^ NK-cells that secrete cytokines. The CD14^+^ CD11c^+^ CD11b^mid^ regulatory populations of DCs increased as well. TNF-α levels significantly decreased, while IL-10 levels increased in patients receiving MSCs compared to patients receiving standard therapy. Therefore, the authors concluded that MSCs would be safe and effective for treating patients with COVID-19 pneumonia, especially for patients with acute events [96].

So far, more than 60 clinical trials of various MSC variants in COVID-19 patients have been registered that shows growing interest in this therapeutic strategy.

One of the perspective treatments of COVID-19 patients is convalescent plasma transplantation. Previously, it was shown that a single infusion of convalescent plasma improved the condition of patients with viral infections such as Ebola, SARS, MERS, and influenza A (H1N9). Transfusion of the convalescent plasma resulted in the decrease of the viral load in the respiratory tract, decrease of serum cytokines, and decrease of the mortality rate in these patients [97,98]. Transfusion of the convalescent plasma to patients with COVID-19 led to the decrease of viral load and improvement of respiratory failure as well. These results suggest that convalescent plasma transfusion may be beneficial in patients infected with SARS-CoV-2 [99]. However, the limited number of patients and the study design cannot establish the potential effectiveness of this treatment, and the reported observations require further evaluation in clinical trials.

The main therapy strategies for SARS-CoV-2 focus on the suppression of the excessive inflammatory reaction. However, an additional consequence of the “cytokine storm” is the development of the immunosuppression that manifests as progressing lymphopenia. Therefore, immune correction should be performed at a certain stage of disease progression to prevent immuno-paralysis and the joining secondary infections. Some concerns are connected with the assumption that immunotherapy in these patients with an over-inflammatory phase of the disease may promote the augmentation of the inflammatory response and exacerbation of the disease. However, the sepsis-like mode of the complications caused by COVID-19 allows the suggestion that cytokine therapy will contribute to normalization of the immune status and decline of secondary infections, which is similar to the immune-suppressive conditions developing with a systemic inflammatory response syndrome in serious trauma and sepsis [100,101]. Similar results were received with IL-7 treatment of HIV-infected patients with generalized infection [102]. IL-15 also has a potential effect of immune correction. However, model experiments revealed the cytokine-related hepatotoxicity [103], and the data of clinical studies showed activation of NK cells and T-killers as well as fever, hypotension, and liver failure, which limits the prospects for IL-15 clinical use [104,105].

Taking into account the pathogenesis of the induced excessive inflammatory immune suppressive reaction in SARS-CoV-2 patients, it seems reasonable to include regulatory cytokine IL-2 in the complex therapy, which is the cytokine that boosts lymphocyte proliferation and activation. Importantly, the regulation of cellular and humoral immunity results largely from IL-2 production. These theoretical assumptions are supported by the results of clinical studies of recombinant IL-2 in patients with immunosuppressive status associated with systemic inflammatory response syndrome caused by sepsis or serious trauma. Indications for immune correction therapy may be progressive lymphopenia (below 900 cells/µL) and a decrease of CD4^+^ Th cells [105].

Therefore, it is necessary to stratify patients on the basis of their immune status and prescribe individual immunotherapy, according to the indications.

Clinical trials of immuno-modifying agents did not register an unfavorable effect in patients with sepsis. The potential of the cytokine therapy for the correction of immunosuppressive status was demonstrated, particularly in case of the prolonged course of the disease [106].

## 7. Conclusions

Dysregulation of the immune system plays an essential or even, which determines the role in the COVID-19 pathogenesis. At the initial stage, virus-infected lung tissue cells are recognized and destroyed by the innate immune effectors γδT-lymphocytes and NK (natural killer) cells (Figure 1). These immune cells express Toll-like receptors (TLRs) that recognize pathogens and activate their effector functions such as cytotoxicity and cytokine production. It is well-established that TLRs recognize conservative pathogen structures, which initiate the innate immune response, and act as sensors for viral RNA. They are expressed by NK cells and γδT-cells as well as by antigen-presenting cells such as macrophages and DCs. The interaction of viral RNA and TLRs triggers signaling pathways that boost the production of cytokines involved in Th1 polarization of CD4^+^ Th cells and the induction of an adaptive immune response [107]. The experimental studies on the Chinese macaques showed that SARS-CoV-2 invades the respiratory mucosa and leads to local infiltration first by lymphocytes and, then, by CD163^+^ macrophages. The latter migrate to the lymphoid organs within three days while transporting not only the fragments of destroyed infected cells for antigen presentation but also viral particles in their vesicles. Therefore, macrophages perform their antigen-presenting function, which differentiate into DCs, and, simultaneously, contribute to the dissemination of the virus and the development of productive infection in lymphoid tissue and the systemic spread of infection [108]. Excessive activation of the innate immune cells and macrophages leads to cytokine over-production and the release of reactive molecules that affect not only virus-infected but also intact cells. MAS is a common reaction in severe coronavirus infections such as SARS, MERS, and COVID-19 that manifests as a “cytokine storm” and sepsis-like symptoms. MAS is characteristic of hemo-phagocytosis and the increase of IL-6 levels and IL-2 receptors, which is described in the above-mentioned viral infections. Since macrophages play an essential role in the induction of excessive inflammatory reaction, we may assume that, in a similar way, by following COVID-19 intoxication, gut-associated lymphoid tissue (GALT), which contains the largest macrophage and T-cell pools, contributes significantly to the developing “cytokine storm.” Activation of GALT mononuclear cells in viral infections is associated with increased LPS (lipopolysaccharides) levels, which is also known as microbial translocation. LPS entering into the circulatory system increases the inflammatory cascade, which closes the vicious circle of the systemic inflammatory response syndrome [109,110]. An exaggerated immune response can lead to generalized vascular permeability. The increased permeability of the vascular walls leads to plasma leakage and, along with blood over-coagulation, can induce DIC-syndrome (disseminated intravascular coagulation/thromboembolic disease syndrome).

Special attention should be paid to the population groups at risk, such as elderly patients and cancer patients with the initially marked symptoms of the immune system dysfunction. Thus, aging is associated with the impairment of the immune regulation, which is manifested as a pro-inflammatory condition characterized by high circulating levels of pro-inflammatory markers such as IL-1, antagonist protein of IL-1 receptor, IL-6, IL-8, IL-13, IL-18, C-reactive protein, IFN-α, IFN-β, TGF-β, TNF, its soluble receptors (members of the receptor superfamily TNF 1A and 1B), and amyloid A [111]. This condition, called “Inflammaging,” can become persistent and long-lasting while building a chronic inflammatory reaction [112]. In case of such an immune status, the “cytokine storm” induced by COVID-19 can lead to fatal consequences. Cancer patients, in particular those in the postoperative period and receiving chemo/radiation therapy, have a high risk of COVID-19 infection. Anti-tumor immunotherapy by immune checkpoint inhibitors (ICI) or CAR-T cells (chimeric antigen receptor, CAR), associated with cytokine release syndrome, enhances the probability of the exaggerated systemic inflammatory response syndrome to COVID-19 intoxication. Therefore, there is a challenge for minimizing the risk of COVID-19 infection while achieving the desired effectiveness of cancer treatment.

Several aspects should be carefully analyzed, including the risk of relapse or disease progression, the estimated endpoints of the therapy, the comorbidities including other patients, and the impact of coronavirus transmission in the local community [113]. In order to meet these requirements, some suggestions can be considered, such as changing therapy regimens and limiting patients’ visits to the hospital. In particular, some authors recommend less intensive treatment regimens in order to replace the Pembrolizumab regimen of 200 mg three-weekly by 400 mg six-weekly [114]. The main therapy strategies for COVID-19 are aimed at suppressing the over-inflammatory response. To achieve the effects, some studies used steroids, even though they showed no desired result. Others included IL-6 receptor inhibitors with a certain clinical effect in small cohorts of patients. Even though IL-6 is an important factor for the induction of the systemic inflammatory response syndrome, it is not the sole one, but a cascade of inflammatory mediators. Therefore, some methods for eliminating cytokines and bioactive molecules of a non-peptide nature can be recommended such as methods of extracorporeal blood purification that proved effective in the treatment of sepsis, including modern selective and non-selective sorbents. The rational for this approach is supported by the data on the clinical effectiveness of plasmopheresis and continuous renal replacement therapy (CRRT) with hemofilters of high sorption capacity. A promising approach for managing the “cytokine storm” can become allogeneic mesenchymal stem cells of the bone marrow or cord blood that have immunosuppressive activity. However, immunosuppressive agents can aggravate immunosuppression caused by COVID-19 that has an inhibiting effect on antimicrobial immunity.

Although the main treatment of the COVID-19 intoxication is focused on neutralizing the excessive inflammatory response, it is worth considering an equally significant problem of the immunosuppressive conditions including immuno-paralysis, which lead to the secondary infection. Immunosuppression develops as a result of lymphocyte apoptosis and lympho-phagocytosis by activated macrophages. It manifests as progressive lymphopenia, mainly due to the decreased Th cell numbers (Figure 1). Th cells produce a range of regulatory cytokines including IL-2 and play a major role in linking adaptive T-cell and B-cell immunity. Therefore, the replacement cytokine-based therapy, with IL-2 stimulating lymphocyte proliferative activity, seems particularly appropriate in patients with COVID-19 during the shift from the hyper-inflammatory to the hypo-inflammatory phase. The indications for immuno-correction in these cases are a progressive decrease of relative and absolute counts of circulating lymphocytes (below 20% and 800 cells/µL, respectively). Low-dose cytokine therapy (0.5–1.0 IU pro dose IL-2), which has an immune stimulating effect, should be used for such an immune correction, as shown in the earlier studies on prevention of deep immunosuppression during treatment of sepsis and serious trauma.

Thus, when choosing a treatment strategy for the immune-mediated complications of coronavirus infection, one has to pass between Scylla and Charybdis, which will ensure not missing the point of the immune silence turning into immuno-paralysis in the fight against the “cytokine storm.”

## References

[1] Drosten S., Günther W., Preiser S., van der Werf H., Brodt S., Becker H., Rabenau M., Panning L., Kolesnikova R.A.M., Fouchier A. (2003). Identification of a novel coronavirus in patients with severe acute 160 respiratory syndrome. N. Engl. J. Med..

[2] Azhar E.I., Hui D.S.C., Memish Z.A., Drosten C., Zumla A. (2019). The Middle East Respiratory162 Syndrome (MERS). Infect. Dis. Clin. N. Am..

[3] De Wit E., van Doremalen N., Falzarano D., Munster V.J. (2016). SARS and MERS: Recent insights into emerging coronaviruses. Nat. Rev. Microbiol..

[4] Peiris J.S., Lai S., Poon L.L.M., Guan Y., Yam L.Y.C., Lim W., Nicholls J., Yee W.K.S., Yan W.W., Cheung M.T. (2003). Coronavirus as a possible cause of severe acute respiratory syndrome. Lancet.

[5] Peiris J.S., Chu C.M., Cheng V.C., Chan K.S., Hung I.F., Poon L.L., Law K.I., Tang B.S., Hon T.Y., Chan C.S. (2003). Clinical progression and viral load in a community outbreak of coronavirus-associated SARS pneumonia: A prospective study. Lancet.

[6] Assiri A., Al-Tawfiq J.A., Al-Rabeeah A.A., Al-Rabiah F.A., Al-Hajjar S., Al-Barrak A., Flemban H., Al-Nassir W.N., Balkhy H.H., Al-Hakeem R.F. (2013). Epidemiological, demographic, and clinical characteristics of 47 cases of Middle East respiratory syndrome coronavirus disease from Saudi Arabia: A descriptive study. Lancet Infect. Dis..

[7] Saad M., Omrani A.S., Baig K., Bahloul A., ElZein F., Matin M.A., Selim M.A., Al Mutairi M., Al Nakhli D., Al Aidaroos A.Y. (2014). Clinical aspects and outcomes of 70 patients with Middle East respiratory syndrome coronavirus infection: A single-center experience in Saudi Arabia. Int. J. Infect. Dis..

[8] Hu Y., Sun J., Dai Z., Deng H., Li X., Huang Q., Wu Y., Sun L., Xu Y. (2020). Prevalence and severity of corona virus disease 2019 (COVID-19): A systematic review and meta-analysis. J. Clin. Virol..

[9] Rockx B., Baas T., Zornetzer G.A., Haagmans B.L., Sheahan T., Frieman M., Dyer M.D., Teal T.H., Proll S., Brand J.V.D. (2009). Early Upregulation of Acute Respiratory Distress Syndrome-Associated Cytokines Promotes Lethal Disease in an Aged-Mouse Model of Severe Acute Respiratory Syndrome Coronavirus Infection. J. Virol..

[10] Binnie A., Tsang J.L., Dos Santos C.C. (2014). Biomarkers in acute respiratory distress syndrome. Curr. Opin. Crit. Care.

[11] Zhou F., Yu T., Du R., Fan G., Liu Y., Liu Z., Xiang J., Wang Y., Song B., Gu X. (2020). Clinical course and risk factors for mortality of adult inpatients with COVID-19 in Wuhan, China: A retrospective cohort study. Lancet.

[12] Chen T., Wu D., Chen H., Yan W., Yang D., Chen G., Ma K., Xu D., Yu H., Wang H. (2020). Clinical characteristics of 113 deceased patients with coronavirus disease 2019: Retrospective study. BMJ.

[13] Wong C.K., Lam C.W.K., Wu A.K.L., Ip W.K., Lee N., Chan I.H.S., Lit L.C.W., Hui D.S., Chan M.H.M., Chung S.S.C. (2004). Plasma inflammatory cytokines and chemokines in severe acute respiratory syndrome. Clin. Exp. Immunol..

[14] Prompetchara E., Ketloy C., Palaga T. (2020). Immune responses in COVID-19 and potential vaccines: Lessons learned from SARS and MERS epidemic. Asian Pac. J. Allergy Immunol..

[15] Bonomi L., Ghilardi L., Arnoldi E., Tondini C.A., Bettini A.C. (2020). A Rapid fatal evolution of Coronavirus disease-19 in a patient with advanced lung cancer with a long-time response to Nivolumab. J. Thorac. Oncol..

[16] Liang W., Guan W., Chen R., Wang W., Li J., Xu K., Li C., Ai Q., Lu W., Liang H. (2020). Cancer patients in SARS-CoV-2 infection: A nationwide analysis in China. Lancet Oncol..

[17] Zhang H., Wang L.-W., Chen Y.-Y., Shen X.-K., Wang Q., Yan Y.-Q., Yu Y., Wu Q., Wang X., Zhong Y. (2020). A multicentre study of 2019 novel coronavirus disease outcomes of cancer patients in Wuhan, China. Medrixv.

[18] Yu J., Ouyang W., Chua M.L.K., Xie C. (2020). SARS-CoV-2 Transmission in Patients With Cancer at a tertiary care hospital in Wuhan, China. JAMA Oncol..

[19] Zhang L., Zhu F., Xie L., Wang C., Wang J., Chen R., Jia P., Guan H., Peng L., Chen Y. (2020). Clinical characteristics of COVID-19-infected cancer patients: A retrospective case study in three hospitals within Wuhan, China. Ann. Oncol..

[20] Stroud C.R., Hegde A., Cherry C., Naqash A.R., Sharma N., Addepalli S., Cherukuri S., Parent T., Hardin J., Walker P. (2017). Tocilizumab for the management of immune mediated adverse events secondary to PD-1 blockade. J. Oncol. Pharm. Pract..

[21] Bersanelli M., Scala S., Affanni P., Veronesi L., Colucci M.E., Banna G.L., Cortellini A., Liotta F. (2020). Immunological insights on influenza infection and vaccination during immune checkpoint blockade in cancer patients. Immunotherapy.

[22] Bersanelli M. (2020). Controversies about COVID-19 and anticancer treatment with immune checkpoint inhibitors. Immunotherapy.

[23] Agarwal S., June C.H. (2020). Harnessing CAR T-cell insights to develop treatments for hyperinflammatory responses in patients with COVID-19. Cancer Discov..

[24] Hrusak O., Kalina T., Wolf J., Balduzzi A., Provenzi M., Rizzari C., Rives S., Carlavilla M.D.P., Alonso M.E.V., Pinilla N.D. (2020). Flash survey on severe acute respiratory syndrome coronavirus-2 infections in paediatric patients on anticancer treatment. Eur. J. Cancer.

[25] Minotti C., Tirelli F., Barbieri E., Giaquinto C., Donà D. (2020). How is immunosuppressive status affecting children and adults in SARS-CoV-2 infection? A systematic review. J. Infect..

[26] Balduzzi A., Brivio E., Rovelli A., Rizzari C., Gasperini S., Melzi M.L., Conter V., Biondi A. (2020). Lessons after the early management of the COVID-19 outbreak in a pediatric transplant and hemato-oncology center embedded within a COVID-19 dedicated hospital in Lombardia, Italy. Bone Marrow. Transplant..

[27] André N., Rouger-Gaudichon J., Brethon B., Phulpin A., Thébault É., Pertuisel S., Gandemer V. (2020). COVID-19 in pediatric oncology from French pediatric oncology and hematology centers: High risk of severe forms?. Pediatr. Blood Cancer.

[28] Fathi N., Rezaei N. (2020). Lymphopenia in COVID-19: Therapeutic opportunities. Cell Biol. Int..

[29] Tan L., Wang Q., Zhang D., Ding J., Huang Q., Tang Y.-Q., Wang Q., Miao H. (2020). Lymphopenia predicts disease severity of COVID-19: A descriptive and predictive study. Signal Transduct. Target. Ther..

[30] Yang X., Yu Y., Xu J., Shu H., Xia J., Liu H., Wu Y., Zhang L., Yu Z., Fang M. (2020). Clinical course and outcomes of critically ill patients with SARS-CoV-2 pneumonia in Wuhan, China: A single-centered, retrospective, observational study. Lancet.

[31] Wang D., Hu B., Hu C., Zhu F., Liu X., Zhang J., Wang B., Xiang H., Cheng Z., Xiong Y. (2020). Clinical characteristics of 138 hospitalized patients with 2019 novel coronavirus-infected pneumonia in Wuhan, China. JAMA.

[32] Liu X., Shen Y., Wang H., Ge Q., Fei A., Pan S. (2016). Prognostic significance of neutrophil-to-lymphocyte ratio in patients with sepsis: A prospective observational study. Mediat. Inflamm..

[33] Moro-García M.A., Alonso-Arias R., López-Larrea C. (2013). When aging reaches CD4+ T-cells: Phenotypic and functional changes. Front. Immunol..

[34] Chiappelli F., Khakshooy A., Greenberg G. (2020). CoViD-19 Immunopathology and Immunotherapy. Bioinformation.

[35] Sakaguchi S., Miyara M., Costantino C.M., Hafler D.A. (2010). FOXP3+ regulatory T cells: In the human immune system. Nat. Rev. Immunol..

[36] Sakaguchi S., Miyara M., Costantino C.M., Hafler D.A. (2000). FOXP3+ regulatory T cells: Key controllers of immunologic self-tolerance. Cell.

[37] Weiskopf M.D., Schmitz S.K., Raadsen M.P., Grifoni A., Okba N.M.A., Endeman H., van den Akker J.P.C., Molenkamp R., Koopmans M.P.G., van Gorp E.C.M. (2020). Phenotype and kinetics of SARS-CoV-2-specific T cells in COVID-19 patients with acute respiratory distress syndrome. Medrxiv.

[38] Qin C., Zhou L., Hu Z., Zhang S., Yang S., Tao Y., Xie C., Ma K., Shang K., Wang W. (2020). Dysregulation of immune response in patients with coronavirus 2019 (COVID-19) in Wuhan, China. Clin. Infect. Dis..

[39] Yu L., Yang F., Zhang F., Guo D., Li L., Wang X., Liang T., Wang J., Cai Z., Jin H. (2018). CD69 enhances immunosuppressive function of regulatory T-cells and attenuates colitis by prompting IL-10 production. Cell Death Dis..

[40] Drosten C., Seilmaier M., Corman V.M., Hartmann W., Scheible G., Sack S., Guggemos W., Kallies R., Muth D., Junglen S. (2013). Clinical features and virological analysis of a case of Middle East respiratory syndrome coronavirus infection. Lancet Infect. Dis..

[41] Zhou Y., Fu B., Zheng X., Wang D., Zhao C., Qi Y., Sun R., Tian Z., Xu X., Wei H. (2020). Aberrant pathogenic GM-CSF+ T cells and inflammatory CD14+CD16+ monocytes in severe pulmonary syndrome patients of a new coronavirus 2020. Medrixv.

[42] Lew T.W., Kwek T.-K., Tai D., Earnest A., Loo S., Singh K., Kwan K.M., Chan Y., Yim C.F., Bek S.L. (2003). Acute Respiratory Distress Syndrome in critically ill patients with Severe Acute Respiratory Syndrome. JAMA.

[43] Sandoval-Montes C., Santos-Argumedo L. (2005). CD38 is expressed selectively during the activation of a subset of mature T cells with reduced proliferation but improved potential to produce cytokines. J. Leukoc. Boil..

[44] Liao S., Xiao S., Zhu G., Zheng D., He J., Pei Z., Li G., Zhou Y. (2014). CD38 is highly expressed and affects the PI3K/Akt signaling pathway in cervical cancer. Oncol. Rep..

[45] Thevarajan I., Nguyen T.H.O., Koutsakos M., Druce J., Caly L., Van De Sandt C.E., Jia X., Nicholson S., Catton M., Cowie B. (2020). Breadth of concomitant immune responses prior to patient recovery: A case report of non-severe COVID-19. Nat. Med..

[46] Channappanavar R., Perlman S. (2017). Pathogenic human coronavirus infections: Causes and consequences of cytokine storm and immunopathology. Semin. Immunopathol..

[47] Min C.-K., Cheon S., Ha N.-Y., Sohn K.M., Kim Y., Aigerim A., Shin H.M., Choi J.-Y., Inn K.-S., Kim J.-H. (2016). Comparative and kinetic analysis of viral shedding and immunological responses in MERS patients representing a broad spectrum of disease severity. Sci. Rep..

[48] Ulhaq Z.S., Soraya G.V. (2020). Interleukin-6 as a potential biomarker of COVID-19 progression. Méd. Mal. Infect..

[49] Ohno Y., Kitamura H., Takahashi N., Ohtake J., Kaneumi S., Sumida K., Homma S., Kawamura H., Minagawa N., Shibasaki S. (2016). IL-6 down-regulates HLA class II expression and IL-12 production of human dendritic cells to impair activation of antigen-specific CD4+ T cells. Cancer Immunol. Immunother..

[50] Huang C., Wang Y., Li X., Ren L., Zhao J., Hu Y., Zhang L., Fan G., Xu J., Gu X. (2020). Clinical features of patients infected with 2019 novel coronavirus in Wuhan, China. Lancet.

[51] Costela-Ruiz V.J., Illescas-Montes R., Puerta-Puerta J.M., Ruiz C., Melguizo-Rodríguez L. (2020). SARS-CoV-2 infection: The role of cytokines in COVID-19 disease. Cytokine Growth Factor Rev..

[52] Lansbury L., Rodrigo C., Leonardi-Bee J., Van-Tam J., Lim W.S. (2019). Corticosteroids as adjunctive therapy in the treatment of influenza. Cochrane Database Syst. Rev..

[53] Chen C., Zhang X.R., Ju Z.Y., He W.F. (2020). Advances in the research of cytokine storm mechanism induced by Corona Virus Disease 2019 and the corresponding immunotherapies. Zhonghua Shao Shang Za Zhi = Chinese J. Burns.

[54] Mogensen S.C., Virelizier J.L. (1987). The IFN-macrophage alliance. Interferon.

[55] Hadchouel M., Prieur A.-M., Griscelli C. (1985). Acute hemorrhagic, hepatic, and neurologic manifestations in juvenile rheumatoid arthritis: Possible relationship to drugs or infection. J. Pediatr..

[56] Nielsen M.J., Andersen C.B.F., Moestrup S.K. (2013). CD163 Binding to haptoglobin-hemoglobin complexes involves a dual-point electrostatic receptor-ligand pairing. J. Boil. Chem..

[57] Bleesing J., Prada A., Siegel D.M., Villanueva J., Olson J., Ilowite N.T., Brunner H.I., Griffin T., Graham T.B., Sherry D.D. (2007). The diagnostic significance of soluble CD163 and soluble interleukin-2 receptor α-chain in macrophage activation syndrome and untreated new-onset systemic juvenile idiopathic arthritis. Arthritis Rheum..

[58] Eloseily E.M., Weiser P., Crayne C., Haines H., Mannion M.L., Stoll M.L., Beukelman T., Atkinson T.P., Cron R. (2020). Benefit of anakinra in treating pediatric secondary hemophagocytic lymphohistiocytosis. Arthritis Rheumatol..

[59] Weiss E.S., Girard-Guyonvarc’H C., Holzinger D., De Jesus A.A., Tariq Z., Picarsic J., Schiffrin E.J., Foell D., Grom A.A., Ammann S. (2018). Interleukin-18 diagnostically distinguishes and pathogenically promotes human and murine macrophage activation syndrome. Blood.

[60] Grom A.A., Horne A., De Benedetti F. (2016). Macrophage activation syndrome in the era of biologic therapy. Nat. Rev. Rheumatol..

[61] Lachmann G., Knaak C., La Rosée P., Spies C., Nyvlt P., Oberender C., Sander L.E., Suttorp N., Müller-Redetzky H. (2019). Hemophagocytic lymphohistiocytosis in unspecific virus infection. Der Anaesthesist.

[62] Crayne C.B., Albeituni S., Nichols K.E., Cron R. (2019). The Immunology of macrophage activation syndrome. Front. Immunol..

[63] Ab-Rahman H.A., Rahim H., Abubakar S., Wong P.-F. (2016). Macrophage activation syndrome-associated markers in severe dengue. Int. J. Med. Sci..

[64] Jiménez-Hernández E., Martínez-Villegas O., Sanchez-Jara B., Martínez-Martell M.A., Hernández-Sánchez B., Loza-Santiaguillo P.D.R., Pedro-Matías E., Arellano-Galindo J. (2016). Epstein-Barr virus-associated hemophagocytic lymphohistiocytosis: Response to HLH-04 treatment protocol. Bol. Med. Hosp. Infant. Mex..

[65] Kim E.A., Lee K.S., Primack S.L. (2002). Viral pneumonias in adults: Radiologic and pathologic findings. Radiographics.

[66] Ksiazek T.G., Erdman D., Goldsmith C., Zaki S.R., Peret T., Emery S., Tong S., Urbani C., Comer J.A., Lim W. (2003). A novel coronavirus associated with Severe Acute Respiratory Syndrome. N. Engl. J. Med..

[67] Yuen K.Y., Chan P.K.S., Peiris M. (1998). Clinical features and rapid viral diagnosis of human disease associated with avian influenza A H5N1 virus. Lancet.

[68] Nicholls J.M., Poon L.L., Lee K.C., Ng W.F., Lai S.T., Leung C.Y., Chu C.-M., Hui P.K., Mak K.L., Lim W. (2003). Lung pathology of fatal severe acute respiratory syndrome. Lancet.

[69] Yao X.H., Li T.Y., He Z.C., Ping Y.F., Liu H.W., Yu S.C., Mou H.M., Wang L.H., Zhang H.R., Fu W.J. (2020). A pathological report of three COVID-19 cases by minimally invasive autopsies. Zhonghua Bing Li Xue Za Zhi = Chin. J. Pathol..

[70] Rossi F., Tortora C., Argenziano M., Di Paola A., Punzo F. (2020). Cannabinoid receptor type 2: A possible target in SARS-CoV-2 (CoV-19) Infection?. Int. J. Mol. Sci..

[71] Guan W.-J., Ni Z.-Y., Hu Y., Liang W.-H., Ou C.-Q., He J.-X., Liu L., Shan H., Lei C.-L., Hui D.S. (2020). Clinical characteristics of coronavirus disease 2019 in China. N. Engl. J. Med..

[72] Shorr A.F., Thomas S.J., Alkins S.A., Fitzpatrick T.M., Ling G.S. (2002). D-dimer correlates with proinflammatory cytokine levels and outcomes in critically ill patients. Chest.

[73] Kyriazopoulou E., Leventogiannis K., Norrby-Teglund A., Dimopoulos G., Pantazi A., Orfanos S.E., Rovina N., Tsangaris I., Gkavogianni T., Botsa E. (2017). Macrophage activation-like syndrome: An immunological entity associated with rapid progression to death in sepsis. BMC Med..

[74] Lukaszewicz A.-C., Grienay M., Resche-Rigon M., Pirracchio R., Faivre V., Boval B., Payen D. (2009). Monocytic HLA-DR expression in intensive care patients: Interest for prognosis and secondary infection prediction. Crit. Care Med..

[75] Jensen I.J., Sjaastad F.V., Griffith T.S., Badovinac V.P. (2018). Sepsis-induced T cell immunoparalysis: The ins and outs of impaired T cell immunity. J. Immunol..

[76] Novi G., Mikulska M., Briano F., Toscanini F., Tazza F., Uccelli A., Inglese M. (2020). COVID-19 in a MS patient treated with ocrelizumab: Does immunosuppression have a protective role?. Mult. Scler. Relat. Disord..

[77] Wu C., Chen X., Cai Y., Xia J., Zhou X., Xu S., Huang H., Zhang L., Zhou X., Du C. (2020). Risk factors associated with acute respiratory distress syndrome and death in patients with coronavirus disease 2019 pneumonia in Wuhan, China. JAMA Intern. Med..

[78] Xu X., Han M., Li T., Sun W., Wang D., Fu B., Zhou Y., Zheng X., Yang Y., Li X. (2020). Effective treatment of severe COVID-19 patients with tocilizumab. Proc. Natl. Acad. Sci. USA.

[79] Hibler B., Markova A. (2020). Treatment of severe cutaneous adverse reaction with tocilizumab. Br. J. Dermatol..

[80] Luo P., Liu Y., Qiu L., Liu X., Liu D., Li J. (2020). Tocilizumab treatment in COVID-19: A single center experience. J. Med. Virol..

[81] Singanayagam A., Glanville N.S., Girkin J.L., Ching Y.M., Marcellini A., Porter J.D., Toussaint M., Walton R.P., Finney L.J., Aniscenko J. (2018). Corticosteroid suppression of antiviral immunity increases bacterial loads and mucus production in COPD exacerbations. Nat. Commun..

[82] Thomas B.J., Porritt R.A., Hertzog P.J., Bardin P.G., Tate M.D. (2014). Glucocorticosteroids enhance replication of respiratory viruses: Effect of adjuvant interferon. Sci. Rep..

[83] Boor P.P., De Ruiter P.E., Asmawidjaja P.S., Lubberts E., Van Der Laan L.J., Kwekkeboom J. (2017). JAK-inhibitor tofacitinib suppresses interferon alfa production by plasmacytoid dendritic cells and inhibits arthrogenic and antiviral effects of interferon alfa. Transl. Res..

[84] Ritchie A., Singanayagam A. (2020). Immunosuppression for hyperinflammation in COVID-19: A double-edged sword?. Lancet.

[85] Monard C., Rimmelé T., Ronco C. (2019). Extracorporeal blood purification therapies for sepsis. Blood Purif..

[86] Ma J., Xia P., Zhou Y., Liu Z., Zhou X., Wang J., Li T., Yan X., Chen L., Zhang S. (2020). Potential effect of blood purification therapy in reducing cytokine storm as a late complication of critically ill COVID-19. Clin. Immunol..

[87] Wang L., Li X., Chen H., Yan S., Li D., Li Y., Gong Z. (2020). Coronavirus disease 19 infection does not result in acute kidney injury: An analysis of 116 hospitalized patients from Wuhan, China. Am. J. Nephrol..

[88] Fu D., Yang B., Xu J., Mao Z., Zhou C., Xue C. (2020). COVID-19 Infection in a patient with end-stage kidney disease. Nephron.

[89] Heinrichs J., Bastian D., Veerapathran A., Anasetti C., Betts B., Yu X.-Z. (2016). Regulatory T-Cell Therapy for Graft-versus-host Disease. J. Immunol. Res. Ther..

[90] Keto J., Kaartinen T., Salmenniemi U., Castrén J., Partanen J., Hänninen A., Korhonen M., Lähteenmäki K., Itälä-Remes M., Nystedt J. (2018). Immunomonitoring of MSC-Treated GvHD patients reveals only moderate potential for response prediction but indicates treatment safety. Mol. Ther. Methods Clin. Dev..

[91] Wilson J.G., Liu K.D., Zhuo H., Caballero L., McMillan M., Fang X., Cosgrove K., Vojnik R., Calfee C.S., Lee J.-W. (2015). Mesenchymal stem (stromal) cells for treatment of ARDS: A phase 1 clinical trial. Lancet Respir. Med..

[92] Zheng G., Huang L., Tong H., Shu Q., Hu Y., Ge M., Deng K., Zhang L., Zou B., Cheng B. (2014). Treatment of acute respiratory distress syndrome with allogeneic adipose-derived mesenchymal stem cells: A randomized, placebo-controlled pilot study. Respir. Res..

[93] Chang Y.S., Ahn S.Y., Yoo H.S., Sung S.I., Choi S.J., Oh W.I., Park W.S. (2014). Mesenchymal Stem Cells for Bronchopulmonary Dysplasia: Phase 1 Dose-Escalation Clinical Trial. J. Pediatr..

[94] Swart J.F., De Roock S., Nievelstein R.A.J., Slaper-Cortenbach I.C.M., Boelens J.J., Wulffraat N.M. (2019). Bone-marrow derived mesenchymal stromal cells infusion in therapy refractory juvenile idiopathic arthritis patients. Rheumatology.

[95] Chen J., Hu C., Chen L., Tang L., Zhu Y., Xu X., Chen L., Gao H., Lu X., Yu L. (2020). Clinical study of mesenchymal stem cell treatment for acute respiratory distress syndrome induced by epidemic influenza A (H7N9) infection: A hint for COVID-19 treatment. Engineering.

[96] Leng Z., Zhu R., Hou W., Feng Y., Yang Y., Han Q., Shan G., Meng F., Meng D., Du S. (2020). Transplantation of ACE2- Mesenchymal stem cells improves the outcome of patients with COVID-19 pneumonia. Aging Dis..

[97] Burnouf T., Radosevich M. (2003). Treatment of severe acute respiratory syndrome with convalescent plasma. Hong Kong Med. J..

[98] Hung I.F.N., To K.K.-W., Lee C.-K., Lee K.-L., Chan K.K.C., Yan W.-W., Liu R., Watt C.-L., Chan W.-M., Lai K.-Y. (2011). Convalescent plasma treatment reduced mortality in patients with severe pandemic influenza A (H1N1) 2009 virus infection. Clin. Infect. Dis..

[99] Duan K., Liu B., Li C., Zhang H., Yu T., Qu J., Zhou M., Chen L., Meng S., Hu Y. (2020). Effectiveness of convalescent plasma therapy in severe COVID-19 patients. Proc. Natl. Acad. Sci. USA.

[100] Döcke W.-D., Randow F., Syrbe U., Krausch D., Asadullah K., Reinke P., Volk H.-D., Kox W. (1997). Monocyte deactivation in septic patients: Restoration by IFN-gamma treatment. Nat. Med..

[101] Nalos M., Santner-Nanan B., Parnell G., Tang B., McLean A., Nanan R. (2012). Immune effects of interferon gamma in persistent *Staphylococcal Sepsis*. Am. J. Respir. Crit. Care Med..

[102] .Levy Y., Sereti I., Tambussi G., Routy J.P., Lelièvre J.-D., Delfraissy J.F., Molina J.M., Fischl M., Goujard C., Rodriguez B. (2012). Effects of recombinant human interleukin 7 on T-cell recovery and thymic output in HIV-infected patients receiving antiretroviral therapy: Results of a phase I/IIa randomized, placebo-controlled, multicenter study. Clin. Infect. Dis..

[103] Guo Y., Luan L., Rabacal W., Bohannon J.K., Fensterheim B.A., Hernandez A., Sherwood E.R. (2015). IL-15 Superagonist–mediated immunotoxicity: Role of NK Cells and IFN-γ. J. Immunol..

[104] Conlon K.C., Lugli E., Welles H.C., Rosenberg S.A., Fojo A.T., Morris J.C., Fleisher T.A., Dubois S.P., Perera L.P., Stewart D.M. (2015). Redistribution, hyperproliferation, activation of natural killer cells and CD8 T cells, and cytokine production during first-in-human clinical trial of recombinant human interleukin-15 in patients with cancer. J. Clin. Oncol..

[105] Lebedev M.F., Gavrilin S.V., Kozlov V.K., Egorova V.N. (2001). The experience of using roncoleukin in the early period of a traumatic disease. Terra Med..

[106] Kiselevskii M.V., Sitdikova S.M., Abdullaev A.G., Shlyapnikov S.A., Chikileva O.I. (2018). Immunosuppression in sepsis and possibility of its correction. Grekov’s Bull. Surg..

[107] De Marcken M., Dhaliwal K., Danielsen A.C., Gautron A.S., Domínguez-Villar M. (2019). TLR7 and TLR8 activate distinct pathways in monocytes during RNA virus infection. Sci. Signal..

[108] Liu L., Wei Q., Nishiura K., Peng J., Wang H., Midkiff C., Alvarez X., Qin C., Lackner A., Chen Z. (2015). Spatiotemporal interplay of severe acute respiratory syndrome coronavirus and respiratory mucosal cells drives viral dissemination in rhesus macaques. Mucosal. Immunol..

[109] Van de Weg C.A., Koraka P., van Gorp E.C., Mairuhu A.T., Supriatna M., Soemantri A., van de Vijver D.A., Osterhaus A.D., Martina B.E. (2012). Lipopolysaccharide levels are elevated in dengue virus infected patients and correlate with disease severity. J. Clin. Virol..

[110] Brenchley J.M., Price D.A., Schacker T.W., Asher T.E., Silvestri G., Rao S., Kazzaz Z., Bornstein E., Lambotte O., Altmann D.M. (2006). Microbial translocation is a cause of systemic immune activation in chronic HIV infection. Nat. Med..

[111] Ferrucci L., Semba R.D., Guralnik J.M., Ershler W.B., Bandinelli S., Patel K.V., Sun K., Woodman R.C., Andrews N., Cotter R.J. (2010). Proinflammatory state, hepcidin, and anemia in older persons. Blood.

[112] Ferrucci L., Fabbri E. (2018). Inflammageing: Chronic inflammation in ageing, cardiovascular disease, and frailty. Nat. Rev. Cardiol..

[113] Waisberg F., Enrico D., Angel M., Chacón M. (2020). Cancer treatment adaptations in the COVID-19 era. JCO Oncol. Pract..

[114] Mayor S. (2020). COVID-19: Impact on cancer workforce and delivery of care. Lancet Oncol..

